# Let's stay together? Intrinsic and extrinsic factors involved in pair bond dissolution in a recolonizing wolf population

**DOI:** 10.1111/1365-2656.12587

**Published:** 2016-09-28

**Authors:** Cyril Milleret, Petter Wabakken, Olof Liberg, Mikael Åkesson, Øystein Flagstad, Harry Peter Andreassen, Håkan Sand

**Affiliations:** ^1^ Faculty of Applied Ecology and Agricultural SciencesHedmark University of Applied SciencesEvenstadN‐2480KoppangNorway; ^2^ Grimsö Wildlife Research StationDepartment of EcologySwedish University of Agricultural SciencesSE‐730 91RiddarhyttanSweden; ^3^ Norwegian Institute for Nature ResearchTungasletta 27485TrondheimNorway

**Keywords:** anthropogenic, *Canis lupus*, extrinsic, inbreeding, intrinsic, pair bond duration

## Abstract

1. For socially monogamous species, breeder bond dissolution has important consequences for population dynamics, but the extent to which extrinsic or intrinsic population factors causes pair dissolution remain poorly understood, especially among carnivores.
2. Using an extensive life‐history data set, a survival analysis and competing risks framework, we examined the fate of 153 different wolf (*Canis lupus*) pairs in the recolonizing Scandinavian wolf population, during 14 winters of snow tracking and DNA monitoring.
3. Wolf pair dissolution was generally linked to a mortality event and was strongly affected by extrinsic (i.e. anthropogenic) causes. No divorce was observed, and among the pair dissolution where causes have been identified, death of one or both wolves was always involved. Median time from pair formation to pair dissolution was three consecutive winters (i.e. approximately 2 years). Pair dissolution was mostly human‐related, primarily caused by legal control actions (36·7%), verified poaching (9·2%) and traffic‐related causes (2·1%). Intrinsic factors, such as disease and age, accounted for only 7·7% of pair dissolutions. The remaining 44·3% of dissolution events were from unknown causes, but we argue that a large portion could be explained by an additional source of human‐caused mortality, cryptic poaching.
4. Extrinsic population factors, such as variables describing the geographical location of the pair, had a stronger effect on risk of pair dissolution compared to anthropogenic landscape characteristics. Population intrinsic factors, such as the inbreeding coefficient of the male pair member, had a negative effect on pair bond duration. The mechanism behind this result remains unknown, but might be explained by lower survival of inbred males or more complex inbreeding effects mediated by behaviour.
5. Our study provides quantitative estimates of breeder bond duration in a social carnivore and highlights the effect of extrinsic (i.e. anthropogenic) and intrinsic factors (i.e. inbreeding) involved in wolf pair bond duration. Unlike the effects of intrinsic and extrinsic factors that are commonly reported on individual survival or population growth, here we provide quantitative estimates of their potential effect on the social unit of the population, the wolf pair.

## Introduction

Population regulation is often described through intrinsic or extrinsic population processes. Species with strong social structures are often more prone to experience some kind of intrinsic population regulation (Odden *et al*. 2014). In such social systems, extrinsic factors (e.g. predation or hunting mortality) may interact with intrinsic factors in such a way that total mortality increases beyond the effect of the actual direct mortality itself (i.e. causing a super‐additive effect) (Milner, Nilsen & Andreassen 2007; Rutledge *et al*. 2010; Andreassen *et al*. 2013; Borg *et al*. 2015). Due to this super‐additive effect, it is essential to understand the mechanisms involved in population regulation, as a few accidental deaths may have a disproportionally large effect on the population. For instance, many threatened large carnivore populations are exposed to human‐caused mortality events. If these species have strong social bonding between members of a social unit, or experience sexually selected infanticide, such human‐caused mortality can result in the social disruption of the group and/or the loss of dependent offspring (Brainerd *et al*. 2008; Rutledge *et al*. 2010; Gosselin *et al*. 2015).

Many species have evolved complex social systems, with a few dominant individuals monopolizing reproduction within the social unit (Macdonald 1983; Jennions & Macdonald 1994; Hatchwell 2009). This is the case for some threatened large carnivore species, from which several have developed a monogamous mating system. In theory, breeders from socially monogamous species repeatedly face the choice of whether to remain together with their current partner or divorce and find another partner. However, lifetime reproductive success of dominant individuals generally increases with the length of their dominance tenure (Hodge *et al*. 2008; Sánchez‐Macouzet, Rodriguez & Drummond 2014). The duration of pair bonds has also been suggested to have positive effects on reproductive performance of socially monogamous species by increasing pair familiarity (Sánchez‐Macouzet, Rodriguez & Drummond 2014).

Maintaining dominance tenure seems, therefore, to be a primary route to gain fitness in socially monogamous species. However, dominance tenure is threatened by a variety of factors that may vary in space. For instance, recolonization and expansion of large carnivore populations into human‐dominated landscapes is often directly affected by human‐caused mortality, for example through legal (hunting and trapping) and illegal (poaching and poisoning) actions (Mech 1995; Persson, Ericsson & Segerström 2009; Liberg *et al*. 2011). Indirect effects of human activities, for example habitat fragmentation, habitat loss (Delibes, Gaona & Ferreras 2001) and geographical or management boundaries (Bischof, Brøseth & Gimenez 2015), are also known to restrict large carnivores distribution, leading to genetic structuring and sometimes inbreeding depression with strong consequences for population viability (Keller & Waller 2002; Liberg *et al*. 2005), and possibly also dominance tenure (Kempenaers, Adriaensen & Dhondt 1998; Sparkman *et al*. 2012). In addition, large carnivore populations are also affected by extrinsic factors, such as food availability (Zedrosser, Dahle & Swenson 2006; Cubaynes *et al*. 2014) or population intrinsic factors, such as intraspecific competition (Cubaynes *et al*. 2014), which has also been found to affect dominance tenure (Hodge *et al*. 2008; Berger *et al*. 2015).

In this article, we used data from a long‐term monitoring programme of a social carnivore population, the wolf (*Canis lupus*) in Scandinavia (Liberg *et al*. 2012) to examine the causes and the length of an important population demographic trait, pair bond duration. The exhaustive genetic and demographic information collected on the recolonizing Scandinavian wolf population (Wabakken *et al*. 2001, 2012; Liberg *et al*. 2012) offers a unique opportunity to better understand the factors involved in pair dissolution in a large carnivore population that is under strong anthropogenic influence (Karlsson *et al*. 2007; Liberg *et al*. 2011).

Specifically, we aimed at dissociating the effect of intrinsic and extrinsic population factors involved in wolf pair dissolution.

1. First, we quantified pair dissolution and causes of pair dissolution and predicted pair dissolution to be mainly caused by extrinsic (i.e. anthropogenic) factors resulting in short wolf pair bond duration (H1).
2. Then, we quantified to which extent population intrinsic and extrinsic characteristics of the pairs explained risk of pair bond dissolution. We hypothesized that spatial variation in extrinsic factors (mainly anthropogenic) explained spatial variation in pair bond duration (H2). Because the population is still in a recolonization phase, with abundant food resources, we further predicted (H3) that there should be no or small effects of population intrinsic factors, such as intraspecific competition, through food availability or wolf density (Mattisson *et al*. 2013). Finally, (H4) we tested the hypothesis that inbreeding (i.e. intrinsic factor) had a negative role in pair bond duration (Kempenaers, Adriaensen & Dhondt 1998) in addition to the inbreeding depression previously observed in this population (Vila *et al*. 2003; Liberg *et al*. 2005).

## Materials and methods

### Study area

**Figure 1:**
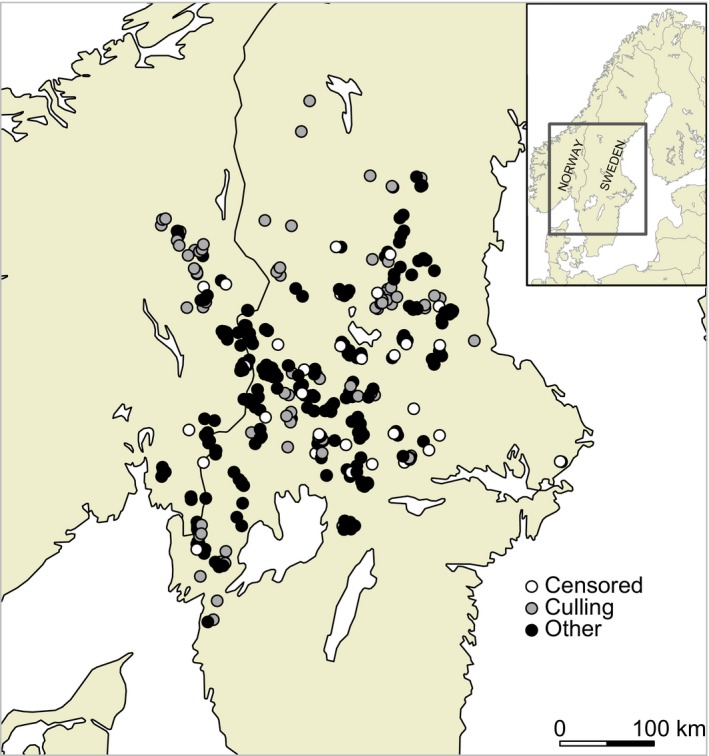
Centroid location of the 369 wolf territorial pair‐winters monitored in Scandinavia during 14 winters, 1998/1999–2011/2012. Grey circles represent pairs that have dissolved due to culling, black circles represent pairs that have dissolved for other reasons, and white circles are for pairs that were censored (i.e. dissolution not observed).

The study was conducted in the south‐central part of the Scandinavian Peninsula (Sweden and Norway 59°–62 °N, 11°–19 °E; Fig. 1). The landscape is dominated by boreal forest, interspersed with bogs and lakes. Agricultural and urbanized lands cover <5% of the study area. Due to extensive commercial logging and forest management practices, the average density of gravel forestry road is high (i.e. 0·88 km km^−2^ inside wolf territories, Zimmermann *et al*. 2014). However, the density of main roads (tarred public roads) is approximately four times lower than the gravel road density (Zimmermann *et al*. 2014). Human density is low and the study area encompasses large areas with less than one human per km^2^ (Wabakken *et al*. 2001). The climate is continental and snow covers the ground for 3–6 months annually, mainly during October–April. Moose are the main wolf prey in Scandinavia and are very abundant (average: 1·3 per km^2^; range 0·7–3·3) throughout the study area (Zimmermann *et al*. 2015).

### Identifying wolf territories and pairs

Monitoring of the Scandinavian wolf population was performed by the Norwegian and Swedish management authorities and consisted of tracking wolves on snow from October 1 to April 30 (Wabakken *et al*. 2001; Liberg *et al*. 2012) over a distance of 2200–5600 km each winter (see further details in Appendix S1, Supporting Information). We also utilized data collected by the cooperative Swedish–Norwegian Wolf Research Project (SKANDULV, Liberg *et al*. 2012) from 3 to 21 territorial wolves equipped with functioning radiocollars each winter. A near complete pedigree of the population has been reconstructed by a combination of individual DNA profiles (samples collected from scats, urine, blood, tissue and hair) and long‐term annual snow tracking of territorial individuals (Liberg *et al*. 2005, 2012; Bensch *et al*. 2006). Information on the spatial location of wolf territories during winter was gathered from snow tracking in combination with DNA‐analyses of collected samples, or by VHF/GPS location when available. The two main goals of the monitoring programme are (i) to register the annual number and spatial distribution of all reproduction events and territorial pairs (hereafter, we used the term territorial wolf pair for: a pair of two potential breeders or two breeders with their offspring, i.e. a pack) and (ii) maintain and continuously update the pedigree of the population. Special tracking efforts were, therefore, made every winter to detect and genetically identify new potential breeders within territorial pairs. This extensive and long‐term monitoring programme provided a near complete description of the annual distribution and dynamics of wolf territories in Scandinavia, including the identities of the territory‐marking individuals (Fig. 1). During our study period (1998/1999–2011/2012), the population increased by fourfold–sixfold from 10 to 60 pairs, and, on average, from 70 to 295 wolves (Wabakken *et al*. 1999, 2012).

### Identifying causes of pair dissolution

All pair dissolutions were assigned to one of five classes: (i) death caused by culling (i.e. legal control actions or license hunting), (ii) verified poaching, (iii) natural causes of death (e.g. age and diseases), (iv) traffic mortality and (v) unknown causes (i.e. when a pair dissolution was verified (one or both individuals were missing), but could not be linked to any of the other four categories). After a pair dissolution event in which one of the pair members went missing, replacement was confirmed when a new wolf started territorial scent marking together with the remaining individual from the previous pair.

### Extracting characteristics of the territory and the pair

In Scandinavia, wolf pair home ranges have an average size of approx. 1000 km^2^ (Mattisson *et al*. 2013). However, accurate home range boundaries (i.e. calculated using at least 9 months with location data, each with five or more locations) were unknown for the majority of pairs, which were not radiocollared (Mattisson *et al*. 2013). Instead, we used all available spatial information (i.e. VHF/GPS and/or snow‐tracking locations) to compute a centroid point location for each territory and year. We then extracted the large‐scale spatial characteristics (Table 1) of the wolf territories within an average circular wolf territory of 1000 km^2^ placed around this centroid point (Mattisson *et al*. 2013; Ordiz *et al*. 2015).

**Table 1:** List of variables used to model wolf risk of pair bond dissolution in Scandinavia during the period 1998–2011. The name, description and related‐hypothesis of each variable used are mentioned. Time series shows whether the variables used varied with time or not. Quadratic effect shows whether a quadratic effect of the variable was tested or not

Name	Description	Hypothesis	Time series	Quadratic effect	Sources
Road1	Total length of paved roads (km per km^2^)	(H2) Reflects human activity	No	No	(1:100 000, Lantmäteriet, Sweden; N50 kartdata, Staten‐skartverk, Norway)
Road2	Total length of gravel roads (km per km^2^)	(H2) Reflects human accessibility	No	No	(1:100 000, Lantmäteriet, Sweden; N50 kartdata Statens Kartverk, Norway)
RoadBuild	Per cent of roads stretches with ≤ 2 buildings per km	(H2) Reflects human accessibility & remoteness	No	No	(Lantmäteriet, Sweden; N50 kartdata, Statens Kartverk, Norway
Hum	Human density, number of inhabitants per km^2^	(H2) Reflects human activity	No	No	www.scb.se, Sweden; www.ssb.no, Norway
Conf1	Dogs depredation events	(H2) Reflects potential for conflicts	No	No	www.rovdjursforum.se, Sweden, www.rovbase.no, Norway
Conf2	Sheep depredation events	(H2) Reflects potential for conflicts	No	No	www.rovdjursforum.se, Sweden, www.rovbase.no, Norway
TimePres	Number of winters that wolf pairs occupied the area	(H2) Increase tolerance through time	Yes	No	Wabakken *et al*. (1999, 2001, 2012)
Country	Country in which the wolf territory was located (Sweden/Norway/Cross‐border)	(H2) Human attitudes towards wolves differ between Sweden and Norway	No	No	Gangaas, Kaltenborn & Andreassen (2013)
LocEast	Location on the longitude scale	(H2) Longitude scale	Yes	Yes	WGS 84/UTM zone 33
LocNorth	Location on the latitude scale	(H2) Latitude scale	Yes	Yes	WGS 84/UTM zone 33
LocCore	Distance from core area of the wolf population	(H2) Effect of management	Yes	Yes	Wabakken *et al*. (1999, 2001, 2012)
Density	Number of wolf territories within a 40 km radius	(H3) Density dependence	Yes	No	Wabakken *et al*. (1999, 2001, 2012)
Moose	Annual number of moose shot per km^2^ used as an index for local moose density	(H3) Food availability	Yes	No	http://www.viltdata.se/, Sweden; www.ssb.no, Norway
Age_F Age_M	Proxy for the minimum age of Female and Male pair members	(H3) Effect of age of pair members	Yes	Yes	Wabakken *et al*. (1999, 2001, 2012)
F_male F_female F	Male, female, potential offspring inbreeding coefficients.	(H4) Inbreeding avoidance	No	No	Liberg *et al*. (2005)

#### Extrinsic characteristics

We used human density (number of inhabitants per km^2^), density of gravel roads and main roads (km per km^2^) and an index that combined information on the spatial location of roads and buildings to quantify areas that were both highly accessible by humans yet remote (Table 1, Appendix S2). Wolf depredation on domestic animals and dogs is an important source of conflict with humans in Scandinavia (Herfindal *et al*. 2005; Liberg *et al*. 2010). We therefore quantified the spatial variation of wolf depredation events for domestic sheep and hunting dogs. (Table 1, Appendix S2).

We used descriptors of the geographical location of the territory, such as longitude, latitude and the distance from the core area of the wolf population (here defined as the annual centre of all estimated centroid points of wolf territories) as additional covariates. As tolerance (Gangaas, Kaltenborn & Andreassen 2013) and management of wolves differs between Sweden and Norway, we also included the country in which wolf pairs were located as a covariate in the models.

To map prey density, we created a moose density index based on harvest density (number of moose harvested per km^2^) at the municipality level in Norway and at the moose management unit (‘älgförvaltningsområde’) level in Sweden. Harvest density has been found to be a robust, but delayed, indicator of spatio‐temporal variation in moose density (Ueno *et al*. 2014). To account for this delay, we used harvest density figures from the year *t *+ 1 to estimate a moose density index in year *t*.

#### Intrinsic characteristics

Local density of wolf pairs was used as a proxy of density‐dependent effects on pair bond duration (Mattisson *et al*. 2013). Each winter, we counted the number of neighbouring territories having their centroid point within a 40‐km‐radius buffer (i.e. two times the radius of a large wolf home range) around the centroid location of each pair.

Human tolerance towards carnivores may sometimes increase with time of coexistence (Zimmermann, Wabakken & Dötterer 2001). Based on wolf monitoring data, the centroid location of each winter territory identified was used as the centre of a 1000‐km^2^ buffer zone (i.e. size of an average wolf home range) and a wolf territory was considered present in pixels covering the buffer. We then created a time series of maps showing the number of winters that territorial wolf pairs had been recorded in each pixel (200 × 200 m, Appendix S2, Supporting information) of the study area since the first wolf pair re‐establishment in 1982 (Wabakken *et al*. 2001).

Because age of the individuals forming the pair can affect pair bond duration, we assigned a year of birth to all individuals. However, due to our extensive data set, we could not assign exact year of birth to all individuals. We therefore estimated a latest possible year of birth (i.e. minimum possible age) to obtain a proxy for the age of individuals forming the pair. The latter was estimated using a combination of multiple sources of information, such as the year of first DNA capture and the last year that the parental pair was known to have successfully reproduced. We also assumed that the individual should be minimum 2 years old before the first detected breeding of the individual, and 1 year before first pairing.

Earlier studies have shown that the level of inbreeding may affect fitness traits among Scandinavian wolves (Liberg *et al*. 2005; Bensch *et al*. 2006). We used the reconstructed pedigree to calculate the individual inbreeding coefficient *f* (Liberg *et al*. 2005), which represents the amount of ancestry shared by parents of an individual (Keller & Waller 2002). To estimate the effect of inbreeding depression on pair bond duration, we used the inbreeding coefficient of the individuals in each pair (i.e. the male and female), and the inbreeding coefficient of their potential offspring as separate variables. Five different Finnish–Russian immigrants that formed a pair were assumed to be outbred (i.e. *f *= 0). For two individuals, with missing pedigree information, we randomly assigned a inbreeding coefficient that was derived from the distribution of inbreeding coefficients calculated from the potential mating of individuals available for mating at the time of birth of the two individuals.

### Pair bond duration

We summarized data from individuals identified during tracking events for each winter. If pair members could not be directly detected during a winter, we used indirect information to confirm their presence, such as the genetic detection of offspring from the non‐detected pair member. These multiple sources of information to confirm presence of pair members were combined with a survival analysis framework to quantify the pair bond duration of territorial wolf pairs. Survival analysis refers to statistical procedures for which the outcome variable of interest is time until an event occurs (Kleinbaum & Klein 2011). In our case, each winter monitoring period (October–April) was set as the time unit. A pair detected within a specific territory during each winter was assumed to have been present during that entire winter, because the exact date of dissolution was unknown in most cases. The dissolution event was attributed to the winter in which one or both of the previously identified individuals were no longer detected within a previously defined territory. Thus, we counted the number of consecutive winters in which a specific pair was identified in its territory from the winter of establishment until the winter in which no signs of one or both individuals were found (i.e. pair dissolution). Hence, if a territorial female and male were found together for three consecutive winters, but not during the fourth winter, we considered that the dissolution occurred at the end of the third winter (i.e. the pair persisted for three consecutive winters and for approximately 2 years). Three different criteria for pair dissolution were used as follows: (i) evidence that one or both individuals were dead, (ii) replacement of one or both individuals by another individual the following winter and (iii) failure to record two scent‐marking individuals in a previously verified territory, despite large tracking efforts. Censoring (when monitoring stops without the event of interest having occurred; Kleinbaum & Klein 2011) only occurred at the end of our study in 2011/2012.

We used a Kaplan–Meier survivor function to quantify the probability that a specific pair will persist over time (Kleinbaum & Klein 2011). It is a step function that decreases from 1 (all wolf pairs are intact at time *t*) towards a minimum value of 0 (when dissolution of all pairs has occurred). To model the relative influence of covariates (Table 1) on risk of pair dissolution, we used semiparametric Cox proportional hazard (CPH) models (Kleinbaum & Klein 2011). These models provide hazard ratios (HR) of covariates on the baseline hazards (instantaneous potential of dissolution) for the event to occur at a time *t* per unit time (Kleinbaum & Klein 2011). We used a counting‐process style input, which allows time‐varying covariates to be used (Fieberg & DelGiudice 2009). Pair members were identified as correlated groups of observations and were clustered in order to obtain robust sandwich variance estimators (Kleinbaum & Klein 2011).

### Cause‐specific pair dissolution

In the case of multiple causes of pair dissolution, a general approach such as Kaplan–Meier is not sufficient because it involves mutually exclusive events in time (i.e. if pair *i* splits up due to cause *k,* it is not available to split up from cause *j*). We therefore estimated specific causes of dissolution using a nonparametric cumulative incidence function estimator (Heisey & Patterson 2006).

To model the impact of covariates (Table 1) on the cause‐specific risk of pair dissolution, we re‐classified causes of pair dissolution into two main categories, (i) culling (i.e. all legal killing, including control and license hunting) and (ii) other causes (i.e. unknown, natural mortality, verified poaching and traffic related). We created the second category because we could not exclude natural mortality, poaching and traffic‐related causes of dissolution from unknown causes of dissolution. We followed methods described by Lunn & McNeil (1995) and Heisey & Patterson (2006) to account for competing risks. We first duplicated the data set as many times as the number of dissolution causes. Then, we used the ‘strata()’ function to compute different baseline hazard functions for each dissolution cause (Therneau 2014). Finally, we included interaction terms between important covariates obtained after model selection and strata to estimate the potential effects of covariates in relation to different causes of dissolution.

### Model selection

To determine which factors (Table 1) influenced risk of pair dissolution, we performed CPH model selection based on corrected Akaike's information criterion (AICc) (Burnham & Anderson 2002; Liang & Zou 2008). Before running model selection, we checked for collinearity between all covariates (*r *< 0·6). Among two correlated variables, only the variable with the lowest AICc score in a simple model was retained in the model selection process (Appendix S3). We standardized all continuous covariates to 1 SD to facilitate interpretation and comparison of the relative strength of parameter estimates (Schielzeth 2010; Grueber *et al*. 2011). All combinations of additive variables were biologically plausible. Therefore, we considered all possible combinations of models (Table 1), using the ‘MuMIn’ r package (Barton 2014). We did not consider individual models with more than five variables to avoid over‐fitting models (Grueber *et al*. 2011). We considered the quadratic forms of some of the variables (Table 1) in the model selection process, but only included that transformation when a model containing both the linear and quadratic forms of the variable had a lower AICc (i.e. ∆AICc ≥ 2) than a model containing just the linear form. We also considered some interactive terms (age_F × age_M; age_F × F_female; age_M × F_male), but only if the interactive model had lower AICc (i.e. ∆AICc ≥ 2) compared to the inclusion of additive model. We then checked for hazard proportionality using the scaled Schoenfeld residuals (Kleinbaum & Klein 2011). We performed model averaging, based on AICc, and calculated confidence intervals for all models with ∆ AICc ≤ 2 (Burnham & Anderson 2002; Grueber *et al*. 2011; Barton 2014). In addition, we used 95% confidence intervals around averaged hazard ratio estimates to help interpret uncertainty in parameters estimation and variable importance (Fletcher & Dillingham 2011; Galipaud *et al*. 2014). Additionally, we also tested whether the replacement of one individual in the pair could be attributed to its degree of relatedness by comparing the inbreeding coefficient of the new individual to the inbreeding coefficient of the replaced individual, using a paired t‐test. We also tested the robustness of our method to extract landscape characteristics, by adding some noise to the centroid location of the territory (See Appendix S4 for further details). All analyses were performed using r version 3.0.3 (R Core Team 2014) and the Survival package (Therneau 2014).

## Results

### Pair dissolution

#### General

**Figure 2:**
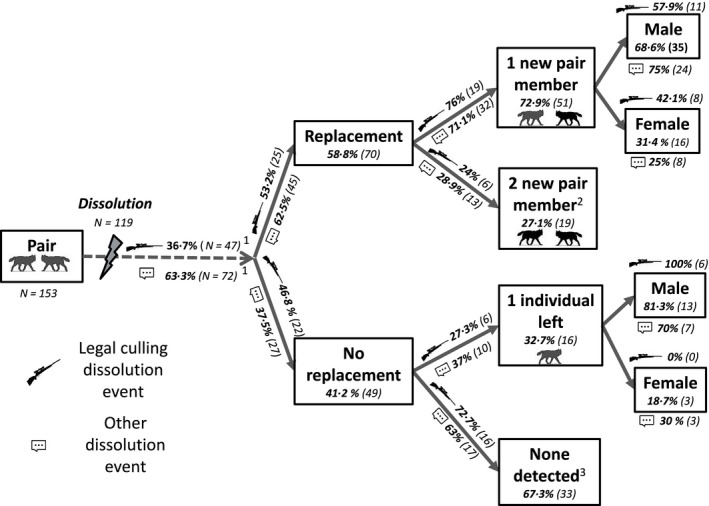
Flow chart of the consequences of pair dissolution in the Scandinavian wolf population during the period 1998–2011. Among the 153 different wolf pairs included in this study, 47 dissolved due to legal culling (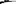) and 72 pairs due to others causes (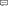; i.e. natural, traffic‐related, poaching and unknown causes). The winter following a dissolution event, we identified either: (i) a replacement of two individuals (i.e. both the male and the female were replaced) or one individual (i.e. the male or the female was replaced); or (ii) no replacement, meaning that we detected one individual left alone (i.e. the male or the female) or no pairs could be confirmed within the territory. Percentages and number of events are presented to show the extent to which culling and other dissolution events were followed by a replacement or not. ^1^Percentages were estimated using nonparametric cumulative incidence function estimator (see methods). ^2^At least one new pair (two new individuals) detected with a territory overlapping the territory of the previously dissolved pair. ^3^No pair could be detected overlapping with the territory previously occupied by the dissolved pair.

Genetic identity of both territorial wolf pair members (i.e. the scent‐marking female and male) was determined in 98% of the 442 winter territories documented during 14 consecutive winters from 1998/1999 to 2011/2012. In total, we detected 179 different pairs representing 429 monitored pair‐winters and 295 different individuals (140 females, 155 males). Among our 13 winter to winter pair bond duration estimates, we determined the fate of 153 different pairs and documented a total of 119 dissolution events. The winter following most of these dissolution events, a replacement occurred for 70 pairs (58·8%), with one (72·9%, *n* = 51) or both pair members (27·1%, *n* = 19) replaced (Fig. 2). For the remaining 49 (41·2%) dissolution events, no replacement occurred before or during the next winter, and we detected one individual being left alone in 32·7% (*n* = 16) of the cases. However, we could not detect any individuals or pairs within the territory previously occupied by the dissolved pair in 67·3% (*n* = 33) of the cases (Fig. 2). Our proxy for minimum age showed that mean (±SD) age at pair establishment was 2 (±1·61) and 2·4 (±1·90) years old, 3·7 (±2·37) and 4·1 (±2·60) at pair dissolution, and mean age of wolves observed in a pair was 3·2 (±2·21) and 3·64 (±2·50) for males and females, respectively.

#### Causes of pair dissolution

**Figure 3:**
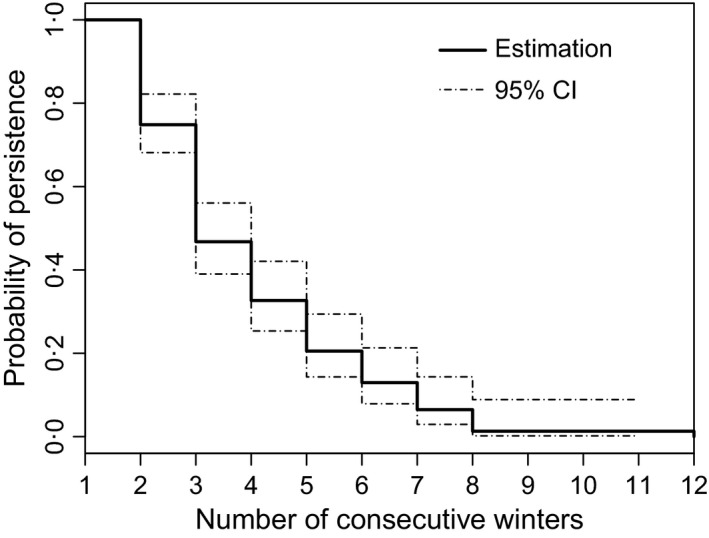
Kaplan–Meier cumulative survival curve with 95% confidence intervals showing the probability of wolf pair bond persistence in Scandinavia during the winters 1998/1999–2011/2012. On the *x*‐axis, winter 1 shows the first winter a pair was detected, winter 2 the second and so on.

Altogether, the survival curve indicated that half of the pairs (i.e. median persistence of pairs) have dissolved after three [95% CI = (3–4)] consecutive winters (i.e. after approximately 2 years) (Fig. 3). The overall probability of a wolf pair bond persisting from one winter to the next (i.e. approximately 1 year) was 0·68 (0·63–0·73). No pair lasted for more than eight consecutive winters, except one that lasted for 12 consecutive winters, with both male and female being at least 13 years old when the pair dissolved. Dissolution due to unknown causes was most common and occurred in 44·3% [95% CI = (37·8–50·8)] of the cases. Causes of dissolution were determined in 55·7% of the cases with 36·7% (25·9–47·5%) of the cases attributed to culling, 9·2% (0–20·1%) to confirmed poaching, 7·7% (0–20·6%) to natural causes of mortality such as disease and age and 2·1% (0–11·9%) to traffic‐related accidents.

### Effect of intrinsic and extrinsic characteristics of the pair on risk of pair dissolution

According to the final CPH models based on all wolf pairs, the extrinsic variables *Distance from the core area (LocCore)* and *Longitudinal gradient (LocEast)*, and intrinsic variables *Inbreeding coefficient of the male (F_male)* and *Age of the males (Age_M*) were the most important variables affecting pair bond duration. The low relative variable importance and 95% confidence interval of hazard ratios not overlapping with 1 (Tables 2 and 3) showed that all other variables had considerably less influence on pair bond duration. Risk of pair dissolution increased with the *Distance from the core area*,* Age of the male* and *Inbreeding coefficient of the male*, and pair bond duration was longer with increasing *longitudinal gradient* (Table 3).

**Table 2:** Model inferences based on Cox proportional hazard regression models of factors affecting risk of pair dissolution in Scandinavia during the period 1998–2011. Best models based on AICc selection and the null model is also presented for comparison purposes. See Table 1 for variable descriptions

Model set	*K*	logLik	AICc	∆AICc	*W*_i_
AgeM + LocCore + LocEast + F_male	4	−475·66	959·37	0	0·27
AgeM + LocCore + LocEast + F_male + AgeF	5	−474·86	959·81	0·43	0·22
AgeM + LocCore + LocEast + F_male + Moose	5	−475·56	961·21	1·83	0·11
AgeM + LocCore + LocEast + F_male + F	5	−475·58	961·25	1·88	0·10
AgeM + Density + LocCore + RoadBuild + LocEast	5	−475·61	961·31	1·93	0·10
AgeM + F_male + LocCore + RoadBuild + LocEast	5	−475·62	961·32	1·94	0·10
AgeM + F_female + F_male + LocCore + LocEast	5	−475·63	961·34	1·97	0·10
Null	1	−485·63	971·26	11·89	0

Only models with ∆ corrected Akaike's information criterion (AICc) < 2 are shown. *K* stands for number of parameters; *W*_i_ for the model weight; logLik for log likelihood.

**Table 3:** Summary of parameter estimates after model averaging the hazard ratios of each parameter on wolf pair bond duration in Scandinavia during the period 1998–2011. A hazard ratio > 1·0 corresponds to an increased risk of pair dissolution for each additional unit of the covariate. All covariates were scaled to 1 SD for comparison purposes. Estimates were calculated from the best models selected after AICc selection (Table 3). See Table 1 for variable descriptions

Parameter	Hazard ratio	95% CI	Relative variable importance
LocCore	1·30	1·10–1·52	1·00
LocEast	0·82	0·70–0·96	1·00
Age_M	1·30	1·09–1·54	1·00
F_male	1·35	1·12–1·63	1·00
Age_F	1·16	0·95–1·42	0·22
Moose	1·05	0·87–1·26	0·11
F_female	0·98	0·84–1·14	0·10
Density	1·03	0·86–1·24	0·10
F	1·04	0·87–1·25	0·10
RoadBuild	0·97	0·81–1·16	0·10

**Figure 4:**
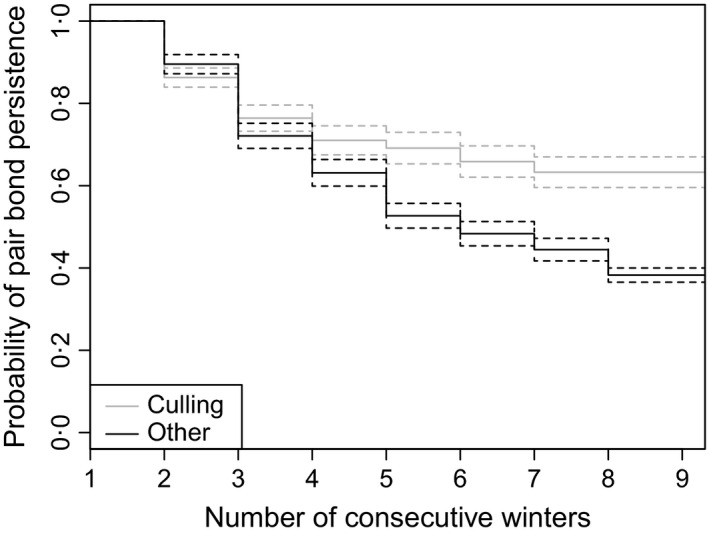
Nonparametric cumulative incidence estimates with 95% confidence intervals showing the probability of wolf pair bond persistence between the winters of 1998/1999 and 2011/2012, which dissolved due to either legal culling in grey (median pair persistence = 3 winters) and all other causes in black (median pair persistence = 4 winters). On the x‐axis, winter 1 shows the first winter a pair was detected, winter 2 the second and so on.

On average, pair dissolution tended to occur earlier when dissolution was due to culling compared to other causes (Fig. 4). The competing risk analysis and the 95% confidence intervals revealed that the risk of pair dissolution found for the intrinsic variables; *Inbreeding coefficient* and *Age of the male* were more important for dissolution due to other causes [HR_F_male _= 1·35, 95% CI = (1·04–1·76); HR_Age_M _= 1·32 (0·99–1·76), respectively] than due to culling [HR_F_male _= 1·38 (0·96–1·98); HR_Age_M _= 1·27 (0·89–1·81), respectively]. Concerning the extrinsic variables, the effect of *Longitudinal gradient* was more important for dissolution due to other causes (HR = 0·77, 95% CI = 0·61–0·97) than due to culling (HR = 0·90, 95% CI = 0·69–1·18). Conversely, the effect of *Distance from the core area* was more important for dissolution due to culling (HR = 1·52, 95% CI = 1·12–2·06) than due to other causes (HR = 1·16, 95% CI = 0·91–1·47).

### Inbreeding coefficient of the new replaced males

Since the males inbreeding coefficient was retained as an important variable, we checked whether inbreeding coefficient of the new male would be lower after a new replacement. However, new males were on average as inbred as the replaced males (average *f* new male = 0·266; average *f* old male = 0·241; *t *= 0·96, d.f. = 24, *P* = 0·35), and consequently, the arrival of a new male in the pair had no effect on the inbreeding coefficient of their pups (average *f* after new male = 0·285; average *f* before new male = 0·298; *t* = −0·54, d.f. = 24, *P* = 0·60).

## Discussion

### Importance of extrinsic factors in causes of pair dissolution

According to our hypothesis (H1), causes of pair bond dissolution were mainly due to extrinsic (i.e. anthropogenic) factors. The death of one or both partners was the typical proximate cause of pair dissolution (Hinton *et al*. 2015), which was supported by data from 98 radio‐marked pair members (Liberg *et al*. 2008). No divorces were observed, that is cases in which both individuals were still alive after a pair dissolution event. The cause of pair dissolution could be determined in 55·7% of the cases, and all involved the death of one or both wolves, most of which were caused by humans (culling: 36·7%, verified poaching: 9·2%, traffic: 2·1%) and 7·7% could be attributed to natural factors. Almost half of the dissolution events could not be assigned to any specific cause. If all or most of the unknown causes of pair dissolution were undetected mortality events, there are only two main possibilities: natural deaths or cryptic poaching. Legal culling is by definition reported in all cases, and it is likely that nearly all un‐intended traffic mortalities are also reported. Liberg *et al*. (2008) showed that natural causes made up 5·5% of all mortality of radiocollared breeding pair members in the Scandinavian wolf population. In our study, natural causes of pair dissolution amounted to 7·7% suggesting that a large proportion of the dissolution events caused by natural causes were detected, assuming GPS collared individuals were a representative sample of the population. As a consequence, a cryptic source of mortality, such as poaching, could be the main explanation for the remaining part of the unknown cases of dissolution. Poaching could, theoretically, be responsible for approximately half of all dissolution events which would be of the same magnitude as individual mortality caused by poaching in Scandinavia (Liberg *et al*. 2011).

We cannot entirely rule out the possibility that false absences, that is pairs that were considered dissolved but were actually intact, might explain a large number of dissolution events due to unknown causes. However, the continuously updated pedigree of the population reconstructed from DNA profiles, in combination with the comprehensive tracking effort (e.g. in the winter 2008/2009 approx. 100 field workers tracked wolves for >5400 km; Liberg *et al*. 2012) mean that very few reproducing pairs could have remained undetected for more than 1 year. Furthermore, the joined annual survival probability of female and male pair members (surv_female_ × surv_male _= 0·82 × 0·77 = 0·63) obtained from GPS collared animals (Liberg *et al*. 2008), falls within the confidence interval of our estimate of winter to winter pair bond duration (0·68; 95% CI: 0·63–0·73). This gives support to the estimates of pair bond duration obtained in our study.

Large carnivore mortality in human‐dominated landscapes is often human‐induced, both in Scandinavia (e.g. Bischof *et al*. 2009 for brown bears, Andrén *et al*. 2006 for lynx, Persson, Ericsson & Segerström 2009 for wolverines), and elsewhere (e.g. Jęodrzejewska *et al*. 1996; Falcucci *et al*. 2009 in Europe, Smith *et al*. 2010 in North America). Although the wolf is the most studied large carnivores, we are only aware of one study explicitly quantifying pair bond duration (Hinton *et al*. 2015). In this study, mean breeding pair bond duration of red wolves (*Canis rufus*) was estimated to 2 years (mean life span of wolf was 3·2 years) and >65% of pair bond dissolutions were caused by anthropogenic factors (Hinton *et al*. 2015). These estimates are comparable with the estimates obtained in our study and are quite different from the long wolf pair bond duration that seems to be perceived for wolf (e.g. Mech 1997). Adult wolf mortality rates are generally low in the absence of human offtake (Creel *et al*. 2015), which suggests that a median pair bond duration of three consecutive winters is relatively short for a long‐lived species such as the wolf (e.g. reported to have reached up to 15 years in the wild Carey & Judge 2000) and may reflect the strong impact of human‐related mortality in this population.

### Importance of spatial variation in extrinsic factors on risk of pair dissolution

Our survival analysis revealed that spatial variation in extrinsic factors was an important factor influencing risk of pair dissolution (H2). However, the geographical location of pairs in Scandinavia better explained pair bond duration than the anthropogenic‐related variables. Although a consensus exists among scientists to apply management and conservation actions at a relevant biological unit, administrative or jurisdiction boundaries are often used as a basis for management decisions (Bischof, Brøseth & Gimenez 2015). According to official policy in both Norway and Sweden, wolves are not allowed to establish in all areas of the peninsula. For example, Scandinavian born wolves that move into the reindeer husbandry area (i.e. covering approximately the northern half of Scandinavia) and outside the specific Norwegian management zone established for breeding wolves (i.e. along the southern Swedish–Norwegian border) are promptly killed legally. As a consequence, the wolf breeding area is constrained to central Scandinavia (Fig. 1) which likely explains the higher risk of mortality due to culling observed at the periphery of the population. Furthermore, a greater tolerance for poaching exists in Norway than in Sweden (Gangaas, Kaltenborn & Andreassen 2013), which could be the causal mechanism to the longitudinal trend found in pair bond dissolution. Therefore, this could suggest that risk of pair dissolution may not be related to spatial variation in anthropogenic characteristics of the landscape, but rather to variation in tolerance towards carnivores and poaching.

### Importance of intrinsic factors on risk of pair dissolution

The Scandinavian wolf population currently suffers from severe inbreeding depression that reduces individual fitness (Liberg *et al*. 2005; Bensch *et al*. 2006). We found a negative effect of the male pair member inbreeding coefficient on pair duration (H4), but only for dissolution events caused by ‘other’ causes. The ‘incompatibility hypothesis’ suggests that the pairing of two individuals that are of intrinsically good quality, but when paired together result in reduced fitness, would benefit from pairing with a new partner (Choudhury 1995). Thus, the replacement of pair members with a new individual resulting in relatively less inbred offspring could be a mechanism reflecting inbreeding avoidance (Choudhury 1995; Sparkman *et al*. 2012). Interestingly, this pattern could not be confirmed in this population, since no cases of divorce were detected (i.e. where both pair members were observed as a new pair after a dissolution event). In addition, replaced males were not less inbred than their predecessor. However, we could not directly test for the ‘incompatibility hypothesis’ since this required explicit data on the reproductive success for each pair, and our monitoring did not provide accurate estimates of litter size but only whether reproduction could be confirmed or not. Once wolf pairs started to reproduce, their subsequent reproduction rate was high with >95% of pairs with a confirmed positive reproductive status (SKANDULV unpublished). However, since the proportion of the genome identical by descent, under some circumstances, can vary substantially among individuals with identical pedigree‐based ancestry (e.g. full siblings), true differences in inbreeding and fitness between individuals may not have been captured entirely by using pedigree information (Kardos, Allendorf & Luikart 2014). An alternative explanation is that inbreeding depression may cause increased mortality of highly inbred males (Keller & Waller 2002). However, there has not been any effect of inbreeding on adult mortality detected in this population so far.

Although the Scandinavian wolf population has increased fourfold to sixfold during our study, we did not find evidence of density‐dependent pair dissolution through an increase in local wolf density or through changes in the density of their main prey (moose), as we hypothesized (H3). This is supported by the lack of home range size response to density‐related factors (Mattisson *et al*. 2013) and, so far, there is only one confirmed observation of intraspecific killing among collared Scandinavian wolves (Liberg *et al*. 2008; Wabakken *et al*. 2009). The age of the male was more important than the age of the female for explaining variation in wolf pair bond duration (Table 3). This could be explained by the fact that males tend to have a generally lower survival rate than females in the population (Liberg *et al*. 2008). In another study, males also showed body mass to decline after approximately 5 years, which could be explained by intense intrasexual competition between males causing weak selection for male longevity (MacNulty *et al*. 2009).

### Consequences of wolf pair dissolution

In a socially monogamous species, the maintenance of the family‐based social structure can have important fitness benefits associated with the adaptive evolution of kinship (Lukas & Clutton‐Brock 2013). For instance, pair bond duration (Sánchez‐Macouzet, Rodriguez & Drummond 2014) and the presence of helpers (Sparkman *et al*. 2011) can have positive effects on reproductive success. Moreover, wolf breeder loss can result in lower pup survival, abandonment of territories, dissolution of social groups (Brainerd *et al*. 2008) or unusual behaviour such as incestuous mating (Vonholdt *et al*. 2008). Although the impact of wolf pair dissolution on population growth is context‐dependent (Brainerd *et al*. 2008; Borg *et al*. 2015), the high dissolution rate observed in our study suggests that extrinsic factors (i.e. anthropogenic) could have an impact on the recolonization of the population and would deserve further attention (Liberg *et al*. 2011). While consequences of human impact on populations usually focuses on numerical response (i.e. population size estimates; but see Rutledge *et al*. 2010), we provided quantitative estimates of anthropogenic influence on the dynamics of the social unit of the population, the wolf pair. Additionally, intrinsically linked population factors, such as the high levels of inbreeding observed in this population, also negatively affect the duration of wolf pair bonds and may contribute to inbreeding depression. The mechanisms behind this result are still unclear and further research could help to distinguish whether inbreeding could act on the divorce rate of pairs or lower the survival of highly inbred males. Identifying sources of spatial variation on estimates of fitness related measures, such as pair bond duration, is strongly needed to understand how intrinsic and extrinsic population factors interact to shape the demography of large carnivore populations. This type of information is also essential to provide appropriate recommendations for a conservation‐oriented management (Falcucci *et al*. 2009; Gaillard *et al*. 2010).

## Data accessibility

Data available from Dryad Digital Repository http://dx.doi.org/10.5061/dryad.242t8 (Milleret *et al*. 2016).

## Supporting information


**Appendix S1.** Summary of winter tracking efforts.
**Appendix S2.** Expanded methods description
**Appendix S3.** Coefficient of correlation between highly correlated covariates (*r* > 0.60).
**Appendix S4.** Test of the robustness of the centroid and buffer method.
