# Supplementary material for: Let's stay together? Intrinsic and extrinsic factors involved in pair bond dissolution in a recolonizing wolf population
Source: J Anim Ecol. 2016 Sep 28;86(1):43–54. doi: 10.1111/1365-2656.12587 (PMC5215671; doi:10.1111/1365-2656.12587)
Supplement: Supplementary file 1 — Appendix S1. Summary of winter tracking efforts. Appendix S2. Expanded methods description Appendix S3. Coefficient of correlation between highly correlated covariates (r > 0.60). Appendix S4. Test of the robustness of the centroid and buffer method. [file JANE-86-43-s001.docx]

**Appendix S1**

We summarized minimum tracking efforts performed each winter during the study period (Wabakken *et al.* 1999; Wabakken *et al.* 2012). These are minimum estimates because only tracking events (actually following a track) were reported. Tracking effort and some opportunistic tracking events were performed and not reported. The lower values for 2003/2004 and 2009/2010 winters correspond to a lack of reporting of kilometers tracked for all tracking events that had occurred in Sweden. For all other winters, kilometers (kms) tracked were recorded for both Sweden and Norway.

**Table S1 Tracking efforts for each winter**

| **Winter** | **Minimum kms tracked** |
| --- | --- |
| 1998/1999 | 2600 |
| 1999/2000 | 2266 |
| 2000/2001 | 3305 |
| 2001/2002 | 3137 |
| 2002/2003 | 2165 |
| 2003/2004 | 501 |
| 2004/2005 | 3328 |
| 2005/2006 | 3732 |
| 2006/2007 | 4291 |
| 2007/2008 | 3560 |
| 2008/2009 | 5487 |
| 2009/2010 | 436 |
| 2010/2011 | 4938 |
| 2011/2012 | 2411 |

**Appendix S2**

To explore the influence of spatial variation in the stability of wolf pair bonds, we used a set of maps describing the large scale spatial characteristics of their territory. We created static maps and time-series grid maps with a fine scale resolution (200m $\times$ 200m) for every spatial descriptor (Table 1).

**Construction of the spatial variables**

Human density was estimated as the number of inhabitants per km^2^ in each municipality. We used the density of gravel and paved roads (Basille *et al.* 2013; Mattisson *et al.* 2013; Zimmermann 2014) as an index of anthropogenic impact in terms of human accessibility. First, we obtained and merged national road maps from Sweden and Norway (Mattisson *et al.* 2013; Zimmermann 2014). Then we calculated the total length of paved and forest roads in km per km^2^ for each map pixel (Zimmermann 2014). To identify areas that were both highly accessible by humans yet remote, we combined information on the spatial location of roads and buildings. Spatial location of buildings (distinction between inhabited and uninhabited could not be made) within settlement areas were availabi.e.,le for Sweden, but not for Norway. Therefore, we calculated the mean number of buildings per km^2^ in Swedish settlements (i.e., 450/km^2^) and randomly generated building densities within Norwegian settlements (i.e. minimum density allowed by definition of Norwegian settlements areas = 400 buildings/km^2^ <http://www.ssb.no>) at the same density as observed for Sweden. We then counted the number of buildings per km of road, within a 500m radius buffer zone along each road stretch. A 500 m buffer was chosen to make sure that most of the buildings identified fell within our buffer. For each wolf territory, we calculated the percentage of area available that was both highly accessible yet remote, defined as the area containing <2 buildings per km of road. This covered 25% of the total area occupied by wolf territories.

**Wolf depredation**

Livestock depredation is one the most important causes of human-large carnivore conflicts (Herfindal *et al.* 2005). In Norway, we used records of wolf depredation events (both ’confirmed‘ and ’supposed‘) concerning sheep (http://www.rovbase.no). In Sweden, we used records of depredation events (https://www.rovdjursforum.se/) in which investigation led to the cause of damage (“besiktigad skadeorsak”) being confirmed, with at least 50% certainty as a carnivore attack and the depredator species being confirmed with at least 50% certainty, as a ’wolf‘. However, the two countries have different sheep farming practices; in Norway sheep are largely left to graze unattended and are free-ranging during the summer grazing season (Zimmermann, Wabakken & Dötterer 2001), while in Sweden sheep are generally kept fenced in and are therefore more protected from predation (Dahle *et al.* 1998). Since we were only interested in the spatial distribution of depredation events and "hot-spot" areas within both countries (independent of the number of attacks in each country) we computed separate maps for Sweden and Norway. We used a kernel density estimator (Worton 1989) to estimate the relative spatial density of all recorded wolf depredation events on sheep during the period 1998-2012, using an average smoothing parameter (h) for each country. Then, we re-scaled values to fall between 0 and 1 by dividing each value obtained by the maximum value observed. This allowed us to conserve the relative intensity of hot-spots depredation areas in each country before merging the two maps.

Wolf attacks on hunting dogs are also an important source of conflicts in Scandinavia (Liberg *et al.* 2010). We used all spatial locations of fatal dog attacks recorded in Sweden and Norway from 1998-2012, and a kernel density estimator (using the ’href‘ method) to create a relative spatial density of wolf attacks on dogs.

**Appendix S3**

**Table S3** shows Spearman coefficient of correlation between highly correlated covariates (r > 0.60). Predictor variables used to run the final models were selected based on the best AICc score, and are shown below in bold. See Table 1 for variable descriptions.

| **Variables** | **Variables** | **r** |
| --- | --- | --- |
| LocNorth | Road1 | -0.78 |
| LocNorth | **RoadBuild** | 0.74 |
| **LocEast** | Road2 | 0.63 |
| Road1 | **RoadBuild** | -0.78 |
| Road1 | Hum | 0.74 |
| **RoadBuild** | Hum | 0.59 |

**Appendix S4**

We tested the robustness of the centroid and buffer methods used to extract geographical landscape characteristics for each territory. Although we performed extensive winter tracking of wolf pairs, we still had uncertainty in the exact home range boundaries. To test whether this could have influenced the values of the extracted landscape characteristics, we randomly added some noise (mean=7.5km, SD=2.5) to the centroid coordinates (i.e., for both X and Y coordinates). This resulted in the creation of new buffers that overlapped, on average, 51.3% (range = 1.5-99%) with the observed buffers. This degree of overlap was in accordance with estimates of inter-annual variability in space use by wolves at the territory level, found in other systems (Uboni *et al.* 2015). We then re-extracted all landscape characteristics as described in the methods section and Table 1 of the main text, and recomputed model selection and model averaging. We repeated this process 100 times and we present the mean Hazard ration and 2.5% and 97.5% quantiles in the Table S4 from all simulations obtained. Hazard ratios obtained using the observed centroid location (Table 3 main text) fall within the 2.5-97.5% quantiles hazard after adding some noise to the centroid location. This shows that errors in the location of the centroid, and the way we determined the landscape characteristics (buffer of 1000km^2^), likely had no influence on our results.

Table S4. Mean, 2.5% and 97.5% quantile hazard ratio (HR) obtained from the model averaging of the 100 simulations after random noise was added to the X and Y coordinates of the centroid points for each wolf territory. The column HRo shows the HR estimates obtained using the observed centroid locations (see Table 3 main text)

| **Parameter** | Mean HR | 2.5 | 97.5 | HRo |
| --- | --- | --- | --- | --- |
| LocCore | 1.31 | 1.27 | 1.42 | 1.30 |
| LocEast | 0.81 | 0.79 | 0.84 | 0.82 |
| Age_M | 1.29 | 1.28 | 1.31 | 1.30 |
| F_male | 1.33 | 1.29 | 1.35 | 1.35 |
| Age_F | 1.17 | 1.16 | 1.18 | 1.16 |
| Moose | 1.05 | 0.97 | 1.12 | 1.05 |
| F_female | 0.98 | 0.96 | 0.98 | 0.98 |
| Density | 1.12 | 0.98 | 1.24 | 1.03 |
| F | 1.05 | 0.97 | 1.12 | 1.04 |
| RoadBuild | 0.97 | 0.89 | 1.05 | 0.97 |

**References**

Basille, M., Van Moorter, B., Herfindal, I., Martin, J., Linnell, J.D.C., Odden, J., Andersen, R. & Gaillard, J.-M. (2013) Selecting Habitat to Survive: The Impact of Road Density on Survival in a Large Carnivore. *PLoS ONE,* **8,** e65493

Dahle, B., Sørensen, O.J., Wedul, E.H., Swenson, J.E. & Sandegren, F. (1998) The diet of brown bears Ursus arctos in central Scandinavia: effect of access to free-ranging domestic sheep Ovis aries. *Wildlife Biology,* **4,** 147-158.

Herfindal, I., Linnell, J.D.C., Moa, P.F., Odden, J., Austmo, L.B. & Andersen, R. (2005) Does recreational hunting of lynx reduce depredation losses of domestic sheep? *Journal of Wildlife Management,* **69,** 1034-1042.

Liberg, O., Aronson, Å., Brainerd, S.M., Karlsson, J., Pedersen, H.C., Sand, H. & Wabakken, P. (2010) Integrating research into management of a recolonizing wolf population – the Scandinavian model. *(Eds.). The World of Wolves: New perspectives on ecology, behaviour and policy* (eds M. Musiani, L. Boitani & P. Paquet). University of Calgary Press.

Mattisson, J., Sand, H., Wabakken, P., Gervasi, V., Liberg, O., Linnell, J.C., Rauset, G. & Pedersen, H. (2013) Home range size variation in a recovering wolf population: evaluating the effect of environmental, demographic, and social factors. *Oecologia,* **173,** 1-13.

Uboni, A., Vucetich, J.A., Stahler, D.R. & Smith, D.W. (2015) Interannual variability: a crucial component of space use at the territory level. *Ecology,* **96,** 62-70.

Wabakken, P., Aronson, Å., Sand, H., Steinset, O.K. & Kojola, I. (1999) The wolf in Scandinavia: Status report of the 1998-1999 winter.(in Norwegian with English summary). **19,** 40-40.

Wabakken, P., Svensson, L., Kojola, I., Maartmann, E., Strømseth, T.H., Flagstad, Ø., Åkesson, M. & Zetterberg, A. (2012) The wolf in Scandinavia and Finalnd: Status report of wolf monitoring in the 2011-2012 winter.(in Norwegian with English summary). **5,** 46-46.

Worton, B.J. (1989) Kernel methods for estimating the utilization distribution in home-range studies. *Ecology,* **70,** 164-168.

Zimmermann, B. (2014) Predatory behaviour of wolves in Scandinavia. Ph.D. Thesis. Hedmark University College, Faculty of Applied Ecology and Agricultural Sciences.

Zimmermann, B., Wabakken, P. & Dötterer, M. (2001) Human-carnivore interactions in Norway: How does the re-appearance of large carnivores affect people attitudes and levels of fear. *Forest Snow and Landscape Research,* **76,** 1-17.
